# Evaluation of Risk Factors for Kidney Function Decline and Chronic Kidney Disease in Living Kidney Donors

**DOI:** 10.3390/jcm14113873

**Published:** 2025-05-30

**Authors:** Aysegul Oruc, Derya Pekin, Ceren Sevinc Kahraman, Hatice Ortac, Elif Gullulu, Cuma Bulent Gul, Abdulmecit Yıldız, Alparslan Ersoy, Mahmut Yavuz, Mustafa Gullulu

**Affiliations:** 1 Department of Nephrology, Bursa Uludag University, Bursa 16059, Türkiye; deryapekin@uludag.edu.tr (D.P.); cerensevinc@uludag.edu.tr (C.S.K.);; 2 Department of Biostatistics, Bursa Uludag University, Bursa 16059, Türkiye; haticeortac21@gmail.com

**Keywords:** kidney transplantation, living kidney donor, donor nephrectomy, renal outcome, glomerular hyperfiltration, protein intake, chronic kidney disease

## Abstract

**Background/Objectives**: Living kidney donors (LKDs) can be at risk of chronic kidney disease (CKD). Several conditions are associated with hyperfiltration, such as solitary kidney, obesity, and high protein consumption. In this regard, we aimed to evaluate the risk factors for kidney function decline in LKDs and the effect of daily protein intake. Methods: Data collected from 220 living kidney donors (mean age: 48.35 ± 12.4 years; 55% female) between 2016 and 2023 were evaluated. One-year and long-term outcomes were evaluated following donor nephrectomy, along with the risk factors for GFR decline and the development of CKD. **Results**: The percentage of eGFR decline was 31.15 (IQR: 19.81–37.5) in the first year and 28.18 (IQR: 18.75–38.16) in the third year after donation. None of the donors had end-stage renal disease during the 31 (IQR: 19–71) months. CKD developed in 31 (23.1%) LKDs. In the first year, the risk factors associated with a decline in eGFR exceeding 30% included male gender (OR: 0.25; 95% CI: 0.11–0.57; *p*: 0.001) and baseline eGFR value (OR: 0.95; 95% CI: 0.92–0.98; *p*: 0.002). At the final visit, the risk factors were male gender (OR: 7.19; 95% CI: 3.06–16.94; *p* < 0.001), age (OR: 1.06; 95% CI: 1.02–1.10; *p*: 0.001), and BMI (OR: 1.12; 95% CI: 1.01–1.23; *p*: 0.030). For the development of CKD, the risk factors were male gender (OR: 0.25; 95% CI: 0.09–0.71; *p*: 0.009) and baseline eGFR (OR: 0.88; 95% CI: 0.83–0.93; *p* < 0.001). No association was observed between daily protein intake and renal outcomes in LKDs following donor nephrectomy. **Conclusions**: In the present study, there was no significant unexpected decline in kidney function in donors in the short-term and the medium-term. Age, BMI, and low basal eGFR values should be carefully considered for LKD evaluation. Furthermore, our findings indicated that protein intake does not substantially impact the GFR. Further controlled studies are required to support our findings.

## 1. Introduction

Living kidney donors (LKDs) are the leading source of renal transplantation in Turkiye. The National Nephrology, Dialysis and Transplantation Registry Report of Turkey indicates that at the end of 2023, 78.32% of recipients with a functioning graft (*n*: 8331) were transplanted from a living donor [1]. A 25–40% rate of decrease in glomerular filtration rate is expected after donor nephrectomy, and LKDs can have a risk of chronic kidney disease (CKD) [2,3]. However, the reported risk of end-stage renal disease (ESRD) development is similar to that of the normal population [4].

As a consequence of having a risk of CKD, emphasis should be placed on the evaluation and care of LKDs. Acceptance of a candidate for donation is decided after detailed analyses and evaluations in accordance with current guidelines. Donor candidates at risk of CKD, such as those with morbid obesity, uncontrolled and complicated diabetes mellitus, and hypertension, have to be excluded [5]. Despite the rigorous nature of the evaluation process, a considerable risk of CKD persists in LKDs following donor nephrectomy [6].

Glomerular hyperfiltration is a compensatory mechanism to overcome the decreased nephron number after donor nephrectomy. This hyperfiltration is a reflection of the renal functional reserve (RFR). The RFR is an answer in physiologic or pathological conditions that allows for increasing the glomerular filtration rate (GFR) of residual nephrons, replacing the lost function and maintaining the whole-organ GFR [7].

High protein intake is associated with a significant increase in GFR [7]. It was indicated that an acute protein load of 1–1.2 g/kg in healthy individuals significantly increases GFR independently of baseline GFR [8]. The presence of a solitary kidney, obesity, and high protein intake facilitates the utilization of RFR. Although using RFR maintains renal function, glomerular hyperfiltration is a well-known cause of glomerulosclerosis. In this case, preventing glomerular hyperfiltration is the main aim of preserving renal function, either through medication or life-changing interventions. Besides advanced medical management, lifestyle changes, weight control, smoking cessation, and nutritional support are essential components for preserving renal function in CKD patients and patients at risk of CKD. It is recommended to avoid high protein intake (>1.3 g/kg body weight/d) in adults with CKD at risk of progression [9].

While LKDs have a risk of CKD due to a decreased number of functioning nephrons, making an effort to preserve renal function is essential [3,6]. Evaluating nutrition and protein consumption habits before donation could provide insights into the potential risks for renal function deterioration. Additionally, proven risk factors of obesity and older age have to be considered before donation. In this regard, we aimed to evaluate the risk factors, including daily protein intake, on kidney function decline in LKDs after donor nephrectomy.

## 2. Materials and Methods

We performed a retrospective study involving 220 LKDs (mean age 48.35 ± 12.4 years, 55% female) who underwent donor nephrectomy between 2016 and 2023 at the Uludag University Department of Nephrology, Transplantation Center. Demographic data and laboratory findings were obtained from the electronic file system.

According to the current guidelines, living donor candidates are comprehensively evaluated using laboratory and imaging analyses before transplantation. Urea and creatinine clearance were calculated for the laboratory evaluation via 24 h urine collection. The accuracy of urine collection was determined by calculating daily creatinine excretion per kilogram of body weight. The total daily creatinine excretion in a normal population averages 10 to 26 mg/kg/day. The quantity excreted has been utilized as an approximate index of the completeness of daily urine collection [10]. Creatinine excretion below 10 mg/kg/day was taken as inaccurate urine collection.

Fourteen donors were excluded because of inaccurate urine collection (*n*: 10) and missing data (*n*: 4). A total of 206 donors were included (mean age: 48.0 ± 12.44 years; 109 females) in this study. Daily protein intake was calculated by the Maroni formula, using biochemical and urinary data obtained from 24 h urinary urea collection [11].

Maroni’s Formula: Protein intake (g/day) = (UUN (g/day)+ (0.031 × weight (Kg)) × 6.25 + proteinuria (g/day).

After donor nephrectomy, one-year and long-term renal outcomes and the associated risk factors for eGFR decline were evaluated. eGFR was calculated using the CKD-EPI formula [12]. The individuals were grouped as lower than 1 g/kg/day and higher than 1 g/kg/day according to daily protein intake. The impact of daily protein intake on renal function was evaluated and compared between the groups. Additionally, patients were grouped according to their first-year and long-term eGFR loss rates (eGFR loss ≥ 30% and < 30%). The groups were compared in terms of the demographic and laboratory findings.

Chronic kidney disease was recently defined as abnormalities of kidney structure or function, present for a minimum of 3 months, with implications for health [9]. According to the criteria of decreased GFR for CKD, a GFR below 60 mL/min per 1.73 m^2^ was defined as CKD. We considered an eGFR value of below 60 mL/min per 1.73 m^2^ as CKD. At their final visit, the donors were grouped as CKD and non-CKD regarding eGFR value. The demographic and baseline laboratory findings were compared between the CKD and non-CKD groups.

All procedures performed in this study involved human participants under the ethical standards of the institutional research committee at which the studies were conducted (approval number 2025-5/1) and with the 1964 Helsinki Declaration and its later amendments or comparable ethical standards.

### Statistics

The normality of continuous variables was assessed using the Shapiro–Wilk test. According to the results of the normality test, variables that conform to a normal distribution were expressed as mean ± standard deviation and variables that do not conform to a normal distribution were expressed as median (with interquartile range [IQR]) values; categorical variables were expressed as *n* (%). In the comparisons between two groups, an Independent Samples t test was used in cases of conformity to a normal distribution, and the Mann–Whitney U test was used in cases of non-conformity to a normal distribution. Categorical variables were compared between groups using Pearson Chi-Square test, Fisher’s exact Chi-Square test, and the Fisher Freeman Halton test. The risk factors affecting the eGFR decline rate of 30% at the first year and last visit, and CKD development after donor nephrectomy were evaluated using a logistic regression analysis. The variables age at donation, DM, HT, gender, BMI, basal eGFR value, and daily protein intake were determined as risk factors. In order to determine the risk factors thought to be effective on eGFR change and CKD development, they were first analyzed by a univariate logistic regression analysis, and the variables that met the *p* < 0.25 criterion after the analysis were included in the multivariate Logistic regression model. A backward selection approach was adopted as the variable selection method. In this study, the backward elimination method was applied for variable selection in the logistic regression model. This method starts with patient-related variables and systematically removes those that are not statistically significant from the model. This approach reduces the risk of removing from the model only variables that are significant in combination with other variables and helps to create a simpler and more meaningful model. When the analysis results were examined, it was found that the logistic regression models obtained in the final step were consistent with the data (Hosmer and Lemeshow test *p* = 0.673 and *p* = 0.175) and that the logistic regression models obtained were also significant (*p* < 0.001). For the statistical analyses, the SPSS (IBM Corp. Released 2017. IBM SPSS Statistics for Windows, Version 25.0. Armonk, NY, USA: IBM Corp.) program was used and the type I error rate was accepted as 5%.

## 3. Results

A total of 206 (mean age 48.0 ± 12.44 years, 52.9% female) donors’ data were retrospectively evaluated. Seventeen (8.3%) donors were hypertensive, and seven (3.4%) were diabetic. The baseline mean body mass index (BMI) of kidney transplant donors was 27.99 ± 4.53 kg/m^2^, eGFR was 103 (IQR 95–112) mL/min, creatinine clearance was 109 (IQR 93–129) mL/min, serum creatinine was 0.74 (IQR 0.65–0.83) mg/dL, proteinuria was s177.5 (IQR 105–235) mg/day, and daily protein intake was 69.9 (IQR 57.9–82) g/day and 0.94 (IQR 0.82–1.09) g/kg/day (Table 1). In terms of relationship to the recipient, 82 (39.8%) were parents, 48 (23.3%) spouses, 48 (23.3%) siblings, 9 (4.4%) children, 11 (5.3%) non-related, and 8 (3.9%) other relatives.

Data from 123 donors were deemed eligible in the first year of transplantation. The serum creatinine and eGFR values at 1 year post-transplant were 1.05 (IQR 0.87–1.24) mg/dL and 69.5 (IQR 61–85) mL/min. The percentage of eGFR loss at the first year of donor nephrectomy was 31.15 (IQR 19.81–37.5).

The donors (*n*: 134) who were followed up for more than 12 months were evaluated for long-term outcomes. The mean follow-up time after donor nephrectomy was 31 (IQR 19–71) months. The serum creatinine and eGFR values at 3 years after donation were 0.99 (IQR 0.82–1.25) mg/dL and 72 (IQR 60–90) mL/min. During this interval, the percentage of GFR loss was 28.18 (IQR 18.75–38.16).

The donors were grouped according to their daily protein intake (<1 g/kg/day and ≥1 g/kg/day). The groups had no significant differences regarding baseline, 1-year, and final-visit serum creatinine and eGFR values. In contrast, calculated creatinine clearance was higher in patients consuming protein more than 1 g/kg/day (121 (IQR 99.5–136) vs. 103.4 (IQR 92–119), *p* < 0.001). BMI was significantly lower in those with higher protein intake (26,72 ± 4.1 vs. 28,80 ± 4.6 kg/m^2^, *p* < 0.001) (Table 2).

Patients were grouped according to their first-year and long-term eGFR percentage change (eGFR loss ≥ 30% and <30%). Regarding the investigation of 30% eGFR decline in the initial year following donor nephrectomy, no statistically significant disparities were observed between the study groups concerning BMI, protein intake, the presence of diabetes mellitus (DM), and hypertension (HT). Whereas donors were older and predominantly male, the basal eGFR value was lower in the group with higher than 30% eGFR decline in the first year after donor nephrectomy (Table 3).

When we compared the donors according to the final-visit eGFR loss rate, those exhibiting a greater than 30% eGFR reduction at the final visit post-donor nephrectomy had a lower baseline eGFR and a higher BMI. Additionally, donors with a decline in eGFR exceeding 30% were older and predominantly male. Daily protein intake, follow-up duration, and presence of DM and HT were similar between the groups (Table 4).

According to the criteria of decreased GFR (GFR < 60 mL/min per 1.73 m^2^) for CKD, 31 donors had an eGFR below 60 mL/min/1.73 m^2^. The donors with CKD were significantly older and dominantly male and had a lower basal eGFR at donation (Table 5).

### Risk Factors for GFR Decline and CKD Development

The likelihood of a decline exceeding 30% in eGFR during the first year after donation was significantly lower in males (OR: 0.25; 95% CI: 0.11–0.57; *p*: 0.001) and in donors with higher baseline eGFR values (OR: 0.95; 95% CI: 0.92–0.98; *p*: 0.002) (Table 6). The long-term risk of a decline exceeding 30% in eGFR was associated with male gender (OR: 7.19; 95% CI: 3.06–16.94; *p* < 0.001), age (OR: 1.06; 95% CI: 1.02–1.10; *p*: 0.001), and BMI (OR: 1.12; 95% CI: 1.01–1.23; *p*: 0.030) (Table 7). Male gender (OR: 0.25; 95% CI: 0.09–0.71; *p*: 0.009) and baseline eGFR (OR: 0.88; 95% CI: 0.83–0.93; *p* < 0.001) were identified as significant factors regarding CKD development (Table 8).

## 4. Discussion

Living kidney donors are susceptible to developing CKD, and as such, a comprehensive evaluation of associated risk factors should be meticulously considered prior to donation. In this context, we evaluated the risk factors associated with renal dysfunction after donor nephrectomy among living donors. Consistent with the previous findings, in our study, there has been no significant unexpected decline in renal function in the donors within both the short and medium terms. Furthermore, we did not find any impact of protein intake on the eGFR after donor nephrectomy, where hyperfiltration is a known compensatory mechanism. Male gender, older age, and higher BMI at donation were found to be associated with higher eGFR decline rates in the long term after donor nephrectomy. However, higher basal eGFR values and male gender were associated with lower risk for CKD development.

The effects of nephrectomy in LKDs have been evaluated in several studies. The number of participants, the presence of a compared group of non-donor individuals, and the analyzed parameters varied among these studies. As a consequence, the identified risk of CKD and renal and patient outcomes differed. Several studies demonstrated a 25–45% decrease in GFR. Basal GFR before donation, gender, and BMI are the factors associated with changes in eGFR over time after donor nephrectomy [2]. In this study, which compares long-term renal function changes between LKDs and the healthy population, it was indicated that eGFR increased by +0.35 mL/min per 1.73 m^2^ per year from 6 weeks post-donation, whereas eGFR showed a steady decline of –0.85 mL/min per 1.73 m^2^ per year in non-donors. The renal compensation in living kidney donors was greatest in the first 2 years and began to plateau after 5 years [2]. In a study that evaluated mortality and lifetime risk of renal failure in LKDs, it was found that the survival rates of kidney donors were similar to those of controls matched by age, sex, and racial or ethnic group. GFR was measured using Iohexol GFR in 255 donors, and aging and high BMI were associated with a GFR of less than 60 mL/min [4]. The study revealed a decline in eGFR of approximately 24% (84.0 ± 13.8 to 63.7 ± 11.9 mL/min/1.73 m^2^) over 12 years. The decline rate per year was 0.49 mL/1.73 m^2^ (95% CI: 0.34 to 0.62), with a greater reduction observed in women (0.60 mL/1.73 m^2^/year (95% CI: 0.43 to 0.78) vs. 0.34 (95% CI: 0.14 to 0.55)) [4].

Obesity is a risk factor for CKD. Although there are contradictory findings regarding the effect of BMI on long-term kidney function following donation, several studies showed that a higher BMI was associated with long-term lower GFR after living kidney donation [2,4,13]. Older age at donation is a common risk factor for a significant decline in GFR following donor nephrectomy, as observed in several studies [2,4,14]. The effect of donor gender on GFR decline has been reported differently between studies. The female gender is generally found to be a risk factor [2,4]. To overcome the conflicting effect of gender on renal outcome, some studies made the analysis using gender-adjusted data [13]. According to our findings, although male gender was not a risk factor for a 30% decline in GFR in the first year, it was found to be an associated risk factor for a 30% decline in GFR in the long term, which was consistent with the study by Massie et al. [15]. Several factors might lead to these contradictory findings. First, we used unadjusted data in our study. Additionally, we determined a definitive rate of 30% for GFR decline both for the first year and long-term after donor nephrectomy. Using gender-unadjusted data and a definitive rate of 30% for GFR decline could explain the conflicting findings about gender in our study.

In our study, the risk factors differed according to the time interval after donor nephrectomy. Older age and higher BMI at donation and male gender were the significant risk factors for higher eGFR decline after donor nephrectomy in the long term. The conflicting findings about the risk factors for GFR decline regarding duration could be associated with the wide range of follow-up durations after nephrectomy.

With respect to comprehensive evaluation and making appropriate decisions for donation, LKDs do not face an increased risk of end-stage renal disease and mortality. In a retrospective cohort that compared 96,217 kidney donors with 20,024 controls regarding ESRD risk, the estimated risk of ESRD at 15 years after donation was significantly higher (30.8 per 10,000 (95% CI, 24.3–38.5) vs. 3.9 per 10,000 (95% CI, 0.8–8.9), *p* < 0.001). However, it was stated that the magnitude of the absolute risk increase was small [6]. Although there is approximately a 30% decline in total GFR after donor nephrectomy, according to large controlled studies, ESRD risks did not significantly increase in LKDs. In our cohort, no subjects developed ESRD. Consistent with the previous findings, the percentage of eGFR loss in the first year was 31.15 (IQR 19.81–37.5) and 28.18 (IQR 18.75–38.16) after a median duration of 31 months following donor nephrectomy. Moreover, CKD developed in 31 (23.3%) donors. Gender and basal eGFR were found to be associated risk factors for CKD development.

Following donor nephrectomy, compensatory hyperfiltration, as a function of RFR, maintains long-term total GFR. Hyperfiltration is a risk factor for progressive kidney damage [16]. High protein intake is found to be associated with kidney damage and CKD risk. One of the mechanisms leading to kidney damage is hemodynamic changes through hyperfiltration [17]. This association was demonstrated in a retrospective cohort study that evaluated the effect of protein intake on calculated single-nephron GFR (SNGFR) in 43 living donors. It was shown that SNGFR was directly associated with estimated protein intake and higher protein intake led to glomerular hyperfiltration [18]. In our study, there was no significant association between daily protein intake amount and basal, 1-year, and 3-year serum creatinine and estimated GFR values. However, basal calculated creatinine clearance was significantly higher in the high-protein intake group, which could be associated with the hyperfiltration impact of higher protein intake. Additionally, we demonstrated that lower protein intake was associated with higher BMI, which can be a risk factor for kidney damage.

As mentioned above, risk factors affecting renal outcome vary according to the structure of the studies. Additionally, our findings demonstrate that risk factors for GFR decline over time and CKD development differ. A decline in GFR is an expected consequence, with evidence indicating that this decline is prominent in the early period following donor nephrectomy and begins to plateau afterwards [2]. In this regard, it is our contention that risk factors for the development of CKD should be accorded greater importance.

Several limitations of our study need to be considered. Firstly, it was a retrospective, non-controlled, cross-sectional study. Secondly, the number of dropouts was significant. In the first year and the further follow-up duration, there were 83 and 72 donor dropouts, respectively. The high number of dropouts might be associated with the relief of donation for a beloved one, and the relatively healthy status of donors. Furthermore, significant loss to follow-up of donors is a common issue stated in other studies [2]. Thirdly, protein consumption was determined by a single measurement, which could be misleading. Consecutive measurements derived from dietary records can provide more accurate results. Furthermore, after donor nephrectomy, dietary habits can change, and we did not have any data on protein consumption after surgery. Additionally, baseline creatinine clearance was found to be associated with protein intake in our cohort. However, we evaluated renal function with the estimated GFR value after donor nephrectomy. Using more sensitive methods for evaluating renal function after nephrectomy should improve our study and could enlighten the impact of protein intake on renal function. However, this is the first study that evaluates the impact of protein intake on renal function in LKDs.

In conclusion, although ESRD risk is not significantly increased in LKDs, a 30% decline is unavoidable following donor nephrectomy. In this regard, we consider that the risk factors of obesity and nutritional habits for CKD should be evaluated and could be managed. Following donor nephrectomy, an average loss of 28.7% in eGFR was observed in comparison to the baseline value of donors in our center. The decline rate was consistent with the literature. Higher protein intake is an associated risk factor for glomerular hyperfiltration. Although basal creatinine clearance was higher in donors consuming higher protein, the basal daily protein intake of donors had no significant effect on GFR during the follow-up period in our study. Age, BMI, and baseline eGFR values should be carefully evaluated in the assessment of LKD for CKD risk. Further controlled studies are required to support our findings. Given the established association between high protein intake and the occurrence of hyperfiltration, the need for long-term controlled studies is paramount to substantiate these findings.

## Figures and Tables

**Table 1:** Demographic and laboratory findings of the donors at donation time.

	All (*n*: 206)
**Gender (M/F), *n* (%)**	97/109 (47.1/52.9)
**Age at donation (year)**	48.0 ± 12.44
**DM, *n* (%)**	7 (3.4)
**HT, *n* (%)**	17 (8.3)
**Basal creatinine (mg/dL)**	0.74 (IQR 0.65–0.83)
**Basal eGFR (mL/min)**	103 (IQR 95–112)
**Basal creatinine clearance (mL/min)**	109 (IQR 93–129)
**Proteinuria (mg/day)**	177.5 (IQR 105–235)
**BMI (kg/m^2^)**	27.99 ± 4.53
**Daily protein intake (g/kg/day)**	0.94 (IQR 0.82–1.09)

M: male, F: female, DM: diabetes mellitus, HT: hypertension, BMI: body mass index, eGFR: estimated glomerular filtration rate.

**Table 2:** Comparison of donors according to daily protein intake.

	Protein Intake <1 g/kg/day (*n* = 126)	Protein Intake ≥1 g/kg/day (*n* = 80)	*p*-Value
**Gender, M/F, *n* (%)**	53/73 (42.1/57.9)	44/36 (55/45)	0.070
**DM, *n* (%)**	9 (7.1)	8 (10)	0.468
**HT, *n* (%)**	5 (4)	2 (2.5)	0.571
**Age at donation (year)**	48.76 ± 12.78	46.80 ± 11.87	0.272
**BMI at donation (kg/m^2^)**	28.80 ± 4.6	26.72 ± 4.1	<0.001
**Basal eGFR (mL/min)**	102 (IQR 93–112)	105 (IQR 96.75–111.5)	0.096
**Creatinine clearance (mL/min)**	103.4 (IQR 92–119	121 (IQR 99.5–136	<0.001
**First-year eGFR (mL/min)**	66 (IQR 58–83)	73 (IQR 63.8–90)	0.114
**Last-visit eGFR (mL/min)**	68.5 (IQR 61–85.5)	74 (IQR 60–92)	0.257
**Follow-up time (months)**	29.5 (IQR 19–69.5)	36 (IQR 19–76)	0.475

M: male, F: female, DM: diabetes mellitus, HT: hypertension, BMI: body mass index, eGFR: estimated glomerular filtration rate.

**Table 3:** Comparison of donors according to a 30% decline in eGFR in the first year after donor nephrectomy.

	ΔeGFRyear < 30% (*n* = 59)	ΔeGFRyear > 30% (*n* = 64)	*p*-Value
**Gender, M/F, *n* (%)**	16/43 (27.1/72.9)	40/24 (62.5/37.5)	<0.001
**DM, *n* (%)**	3 (5.1)	2 (3.1)	0.582
**HT, *n* (%)**	3 (5.1)	7 (10.9)	0.235
**Age (year)**	44.54 ± 12.62	52.64 ± 11.21	<0.001
**BMI (kg/m^2^)**	27.77 ± 4.74	28.25 ± 4.05	0.269
**Basal eGFR (mL/min)**	106 (IQR 96.5–115)	97.1(IQR 89.5–103)	<0.001
**Protein intake (g/kg/day)**	0.95 (IQR 0.85–1.12)	0.93 (IQR 0.81–1.04)	0.242

M: male, F: female, DM: diabetes mellitus, HT: hypertension, BMI: body mass index, eGFR: estimated glomerular filtration rate.

**Table 4:** Comparison of donors according to 30% percentage of eGFR decline at the last visit.

	ΔeGFRlast < 30% (*n* = 70)	ΔeGFRlast > 30% (*n* = 64)	*p*-Value
**Gender, M/F, *n* (%)**	17/53 (24.3/75.7)	43/21 (67.2/32.8)	<0.001
**Follow-up time (months)**	31 (IQR 19–74)	30.5 (IQR 19–69)	0.562
**DM, *n* (%)**	2 (2.9)	3 (4.7)	0.577
**HT, *n* (%)**	5 (7.1)	6 (9.4)	0.638
**Age (year)**	44.13 ± 12.26	53.43 ± 10.92	<0.001
**BMI (kg/m^2^)**	27.35 ± 4.41	28.76 ± 4.36	0.033
**Basal eGFR (mL/min)**	106 (IQR 95–115.1)	98.5 (IQR 90–103)	<0.001
**Protein intake (g/kg/day)**	0.95 (IQR 0.85–1.09)	0.93 (IQR 0.80–1.05)	0.343

M: male, F: female, DM: diabetes mellitus, HT: hypertension, BMI: body mass index, eGFR: estimated glomerular filtration rate.

**Table 5:** Comparison of donors with and without a decreased eGFR below 60 mL/min/ 1.73 m^2^.

	CKD (*n* = 31)	Non-CKD (*n* = 103)	*p*-Value
**Gender, M/F, *n* (%)**	22/9 (71/29)	38/65 (36.9/63.1)	<0.001
**Follow-up time (months)**	30 (IQR 22–68)	31 (IQR 19–72)	0.910
**DM, *n* (%)**	1 (3.2)	4 (3.9)	0.865
**HT, *n* (%)**	4 (12.9)	7 (6.8)	0.277
**Age (year)**	56.79 ± 8.78	46.10 ± 12.42	<0.001
**BMI (kg/m^2^)**	27.12 (IQR 26–30.8)	28.6 (IQR 24.5–30.4)	0.675
**Basal eGFR (mL/min)**	90 (IQR 80–99.5)	105 (IQR 96.2–114)	<0.001
**Protein intake (g/kg/day)**	0.95 (IQR 0.83–1.12)	0.94 (IQR 0.83–1.07)	0.694

M: male, F: female, DM: diabetes mellitus, HT: hypertension, BMI: body mass index, eGFR: estimated glomerular filtration rate.

**Table 6:** Logistic regression analysis of risk factors predicting a decline of more than 30% in eGFR in the first year after donation.

	Univariate Logistic Regression Model		Multivariate Logistic Regression Model	
	OR (95% CI)	*p*	OR (95% CI)	*p*
**Age**	1.06 (1.03–1.09)	**0.001**		
**Gender (male)**	4.48 (2.08–9.63)	**<0.001**	0.25 (0.11–0.57)	**0.001**
**BMI (kg/m^2^)**	1.03 (0.95–1.11)	0.536		
**DM**	0.60 (0.09–3.74)	0.586		
**HT**	2.29 (0.56–9.31)	**0.246**		
**Basal eGFR (mL/min)**	0.95 (0.92–0.98)	**<0.001**	0.95 (0.92–0.98)	**0.002**
**Daily protein intake (g/kg/day)**	0.33 (0.06–1.84)	**0.206**		

OR: Odds ratio (odds oranı), CI, confidence interval. DM: diabetes mellitus, HT: hypertension, BMI: body mass index, eGFR: estimated glomerular filtration rate.

**Table 7:** Logistic regression analysis of risk factors predicting a decline of more than 30% in eGFR at last visit.

	Univariate Logistic Regression Model		Multivariate Logistic Regression Model	
	OR (95%CI)	*p*	OR (95%CI)	*p*
**Age**	1.07 (1.04–1.11)	**<0.001**	1.06 (1.02–1.10)	**0.001**
**Gender (male)**	6.38 (2.99–13.59)	**<0.001**	7.19 (3.06–16.94)	**<0.001**
**BMI (kg/m^2^)**	1.08 (0.99–1.17)	**0.068**	1.12 (1.01–1.23)	**0.030**
**DM**	1.67 (0.27–10.34)	0.580		
**HT**	1.35 (0.39–4.64)	0.639		
**Basal eGFR (mL/min)**	0.95 (0.92–0.97)	**<0.001**		
**Daily protein intake (g/kg/day)**	0.35 (0.07–1.79)	**0.208**		

OR: Odds ratio (odds oranı), CI, confidence interval. DM: diabetes mellitus, HT: hypertension, BMI: body mass index, eGFR: estimated glomerular filtration rate.

**Table 8:** Logistic regression analysis of risk factors predicting CKD development.

	Univariate Logistic Regression Model		Multivariate Logistic Regression Model	
	OR (95%CI)	*p*	OR (95%CI)	*p*
**Age**	1.09 (1.05–1.15)	**<0.001**		
**Gender (male)**	4.18 (1.75–10.01)	**0.001**	0.25(0.09–0.71)	**0.009**
**BMI (kg/m^2^)**	1.05 (0.96–1.15)	0.306		
**DM**	0.83 (0.09–7.67)	0.866		
**HT**	2.03 (0.55–7.46)	0.285		
**Basal eGFR (mL/min)**	0.88 (0.84–0.93)	**<0.001**	0.88(0.83–0.93)	**<0.001**
**Daily protein intake (g/kg/day)**	1.46 (0.23–9.11)	0.689		

OR: Odds ratio (odds oranı), CI, confidence interval. DM: diabetes mellitus, HT: hypertension, BMI: body mass index, eGFR: estimated glomerular filtration rate.

## Data Availability

The data presented in this study are available on request from the corresponding author.

## Abbreviations

The following abbreviations are used in this manuscript:

CKD	Chronic kidney disease
LKD	Living kidney donor
ESRD	End-stage renal disease
RFR	Renal functional reserve
GFR	Glomerular filtration rate
IQR	Interquartile range
BMI	Body mass index
DM	Diabetes mellitus
HT	Hypertension
